# Why Brain Oscillations Are Improving Our Understanding of Language

**DOI:** 10.3389/fnbeh.2019.00190

**Published:** 2019-08-22

**Authors:** Antonio Benítez-Burraco, Elliot Murphy

**Affiliations:** ^1^Faculty of Philology, University of Seville, Seville, Spain; ^2^Division of Psychology and Language Sciences, University College London, London, United Kingdom

**Keywords:** oscillations, gamma, delta, theta, cross-frequency coupling, schizophrenia, autism, Neanderthals

## Abstract

We explore the potential that brain oscillations have for improving our understanding of how language develops, is processed in the brain, and initially evolved in our species. The different synchronization patterns of brain rhythms can account for different perceptual and cognitive functions, and we argue that this includes language. We aim to address six distinct questions—the What, How, Where, Who, Why, and When questions—pertaining to oscillatory investigations of language. Language deficits found in clinical conditions like autism, schizophrenia and dyslexia can be satisfactorily construed in terms of an abnormal, disorder-specific pattern of brain rhythmicity. Lastly, an eco-evo-devo approach to language is defended with explicit reference to brain oscillations, embracing a framework that considers language evolution to be the result of a changing environment surrounding developmental paths of the primate brain.

## What: The Oscillatory Nature of Language

During the last 150 years, neurolinguistic research has mostly focused on mapping language to the brain. The advent of various neuroimaging facilities (MRI, EEG/MEG, PET) has allowed neurolinguists to draw precise maps of the “language-ready” brain (that is, our species-specific brain configuration that allows us to learn and use language), both in pathological and neurotypical populations. It is now evident that language results from the coordinated activity of several widespread brain networks, encompassing different areas of both hemispheres (e.g., Poeppel et al., 2012; Chai et al., 2016, among many others). Nonetheless, as Poeppel (2012) has often stated, “mapping is not explaining.”

Research into neural oscillations can allow us to circumvent this crucial limitation of neurolinguistics and provide robust, motivated explanations of how the brain processes language. Oscillations enable the construction of coherently organized neuronal assemblies through establishing transitory temporal correlations. They reflect synchronized fluctuations in neuronal excitability and are grouped by frequency, with the most common rhythms being delta (δ: ~0.5–4 Hz), theta (θ: ~4–8 Hz), alpha (α: ~8–12 Hz), beta (β: ~12–30 Hz) and gamma (γ: ~30–150 Hz). These are generated by various cortical and subcortical structures and form a hierarchical structure. For example, slow rhythms can phase-modulate the power of faster rhythms (see Buzsáki and Draguhn, 2004; Buzsáki and Watson, 2012).

There are many reasons why oscillations are a promising candidate with respect to addressing Poeppel’s (2012) mapping problem. For instance, they are primitive components of brain function and appear to be both domain-general (i.e., individual oscillations intervene in different cognitive and perceptual functions) and domain-specific (i.e., there exists a specific pattern of coupling between oscillations related to, and explaining, each cognitive function); an observation clearly grounded by Başar and Stampfer (1985), Başar (2006) and Güntekin and Başar (2016; see also Hancock et al., 2017; Murphy, 2018). Importantly, the different “grammars” or “neural syntax” (Buzsáki and Watson, 2012) of brain rhythms accounting for different perceptual and cognitive functions are believed to be species-specific, but the atoms encompassing these grammars (i.e., individual rhythms) are shared across many species (Buzsáki et al., 2013; Brincat and Miller, 2015; Esghaei et al., 2015; Başar and Düzgün, 2016; Kikuchi et al., 2017; Murphy and Benítez-Burraco, 2018b). This circumstance grants a noteworthy evolutionary continuity to cognitive functions, which is particularly important in the case of language; meaning, certain elementary computational processes seem to be realized by brain oscillations (e.g., representational merging, working memory processes like search and maintain, and the coordination of distinct memory buffers; Murphy, 2018), and as such small tweaks to their phasal and coupling properties can yield modifications to their scope and format. This helps us cover the “What” question of our target of inquiry; namely, what the object of neurobiological inquiry is with respect to the implementational basis of language as conceived as a computational system. The remaining sections will cover some other questions surrounding the neural implementation of language, argue for a particular oscillatory model of language, and uniquely cover a large number of domains which bear some form of relation to the central theme of brain oscillations. Due to the rapidly expanding size of neurolinguistic research into oscillations, our discussion will include a selective overview of current themes in the literature.

## How: Oscillations and the Linguistic Brain

As also discussed extensively by Poeppel (e.g., Poeppel and Embick, 2005), current neurolinguistic research suffers from two crucial shortcomings. On the one hand, it relies on broad distinctions between components of language (e.g., the syntactic rules of grammar vs. the meanings of lexical representations), which actually involve multiple neural components, computations and representations. On the other hand, the core elements of linguistic theory (e.g., syntactic operations) do not map onto core neurobiological elements (neurons, nodes of Ranvier, etc.). It is consequently urgent for any neurolinguistic research to formulate a model of language in computational terms that can be processed by specific parts of the brain in real-time. For instance, we can decompose syntax into its constituent operations (MERGE, Labeling, Search; Adger, 2019) and representations [lexical and categorial features, such as N(oun) and A(djective); Adger and Svenonius, 2011]. Which seem generic enough to potentially make contact with certain neurobiological information processing frameworks. To take only the most commonly discussed cases, MERGE in its current formulation (Chomsky et al., Forthcoming) involves adding objects to a workspace, while Labeling involves attributing to a constructed set within a workspace a particular categorial identity.

**Figure 1:**
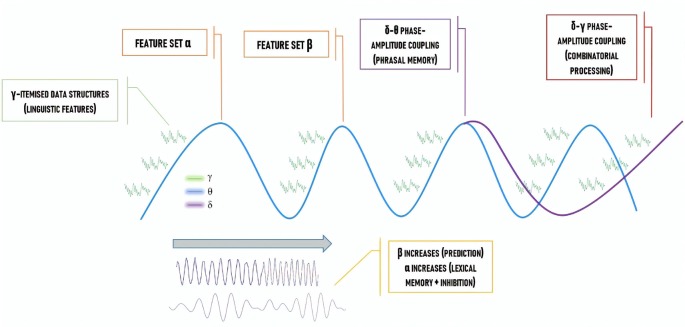
The “How” Question: a neural code for language, representing the various cross-frequency coupling interactions proposed to implement hierarchical phrase structure building.

Decomposing language into a specific pattern of “coupling” between different oscillations (whereby one feature of an oscillation, such as its phase, has its firing pattern synchronized with a feature of a distinct oscillation, such as its amplitude) appears feasible. Importantly, this approach satisfactorily accounts for core facets of language according to major linguistic theories, in particular, generative theories. For instance, the combinatorial power of MERGE (the basic operation in the modern generative approach to language, which adds an object to a given workspace) and the cyclic power of Labeling (the operation which chooses the lexical features to be assigned to the merged syntactic set) are able to be implemented/indexed *via* various oscillatory interactions such as forms of “cross-frequency” (i.e., between distinct frequencies) coupling (Murphy, 2015, 2018; Meyer, 2018). In the most recent and comprehensive oscillatory model of language comprehension defended in Murphy (2016, 2018) and which is briefly summarized in Figure 1, empirical and conceptual motivations are presented to defend the idea that δ-θ inter-regional phase-amplitude coupling constructs multiple sets of linguistic syntactic and semantic features. This occurs when the phase of δ is synchronized with the amplitude of θ. Causal directionality remains an open issue, though certain cases of θ-γ coupling appear to exhibit unidirectional prefrontal-hippocampal cortex coupling from γ activity to θ activity (Nandi et al., 2019). The full computational power of our model is achieved *via* distinct β and γ sources also being coupled with θ (e.g., θ-γ phase-amplitude coupling) for, respectively, syntactic prediction and conceptual binding. This framework goes considerably beyond the discussion of combinatorics, representational accommodation, and prediction presented in other recent accounts (e.g., Meyer, 2018). It also provides a specific neural code for *recursive hierarchical phrase structure*, the core distinctive feature of human language (reapplying the set-forming operation MERGE to its own output), with α also being involved in the early stages of binding (Pina et al., 2018) to synchronize distant cross-cortical γ sites required for the “θ-γ code” (θ-γ phase-amplitude coupling) of working memory and to modulate attentional resources (Figure 1).

**Figure 2:**
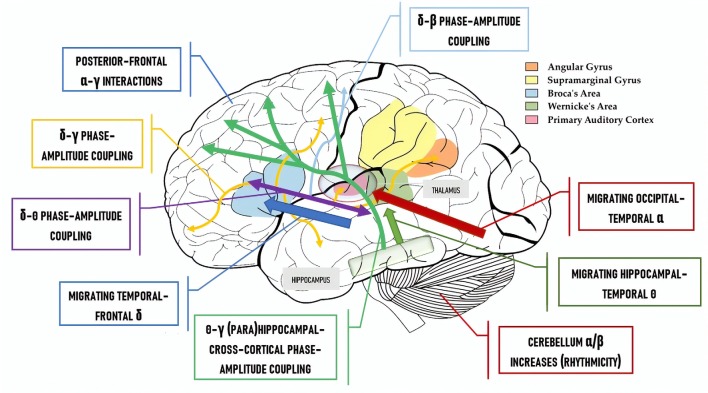
The “Where” Question: a cartographic map of where the neural code for language is hypothesized to be implemented.

Figure 2 contrasts the classical “language areas” with the model we are defending, revealing a considerably greater degree of complexity. To illustrate this point further, Murphy (2018) discusses the high likelihood that traveling oscillations are involved in language comprehension. These are oscillations which “move” across the brain; meaning, the spiking of neural clusters is coordinated not just across two fixed points (e.g., hippocampus and left inferior frontal cortex inter-regional phase-amplitude coupling) but across a particular extended path. These traveling oscillations have recently been shown to coordinate neural activity across widespread brain networks and across different temporal windows, and to support brain connectivity and function (Zhang et al., 2018). Accordingly, under the model in Figure 2, δ waves cycle across left inferior frontal parts of the cortex, building up the syntactic workspace phrase-by-phrase and potentially being endogenously reset by a newly constructed phrase, and being coupled to traveling θ waves which perform the same function (Figure 2; blue arrow and purple box). Traveling δ waves are assumed to be responsible for patterning spiking from single- to multi-unit lexical structures in each δ cycle. As such, δ would coordinate phrasal construction while θ-γ interactions (green arrows) would support the representational construction of linguistic feature-sets (Covington and Duff, 2016; Ding et al., 2016). Lastly, as Gągol et al. (2018) reveal, δ-γ coupling is involved in fluid intelligence (solving problems using a range of cognitive faculties on the fly, spontaneously), whereby δ embeds cross-cortical γ rhythms depending on the cortical areas needed for the particular task, i.e., geometric reasoning, visual processing, etc. Murphy (2018) proposes that δ-γ coupling may be a generic combinatorial process, combining representations from within and across domains (Figure 2; yellow box and yellow arrows), and the cerebellum has also been shown to play a role in processing linguistic rhythmicity and hence aids phrasal processing in frontotemporal regions (Murphy, 2019). Meanwhile, linguistic prediction seems to be implemented *via* coupling between frontal γ amplitude and posterior α phase (Wang et al., 2018) and prefrontal predictions facilitate δ-entrained speech tracking in anterior superior temporal gyrus (Keitel et al., 2017).

Although we refer the reader to Murphy (2018) for further empirical details, we should briefly mention that there is increasing support for this model. For instance, Brennan and Martin (2019) analyzed a naturalistic story-listening EEG dataset and showed that δ-γ coupling increases with the number of predicates bound on a given word (the authors only analyzed the central Cz electrode, so further analysis is required to flesh out the picture). They also discovered an increasing scale of δ-θ coupling beginning at the point of a word completing a single phrase, through to words completing two and three phrases. As such, δ-γ and δ-θ coupling increases with predication. Overall, these observations illustrate how the presently defended analysis of interacting, traveling waves can help explain how such a complex thing as a fragment of discourse, which entails both linguistic and extralinguistic (i.e., encyclopaedic) knowledge, is processed.

## Where: A Systems Biology Approach to Language

Mastering a language and being able to use it depends on having received the proper triggering environmental stimuli during development. But this is only possible because of complex biological processes, which are assembled mostly under genetic guidance. Thousands of biological factors interact to regulate language development and processing. Nevertheless, for many years it was not clear where the specificity of language resides—and if there is much biologically specific at all. Accordingly, although language seems to be a very specialized, human-specific faculty, it undoubtedly relies on biological components, such as its genetic basis, which may not be specific to language since “language genes” contribute to a range of biological functions.

**Figure 3:**
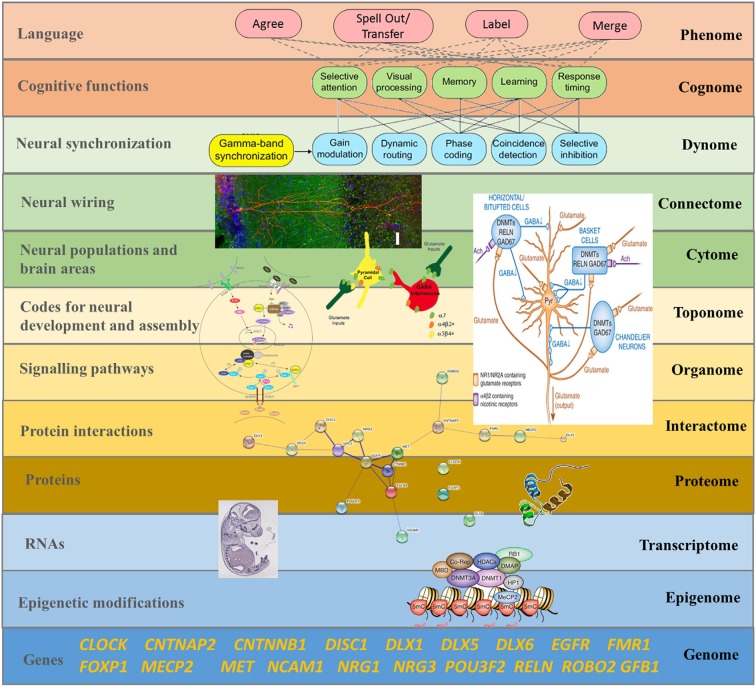
The “Who” Question: a systems biology approach to language, focused on the dynamics of cellular and organismal function and on the (emergent) properties of the whole system, is suggested if one wants to understand how language emerges from these complex interactions (reproduced from Murphy and Benítez-Burraco, 2017; Figure 8).

**Figure 4:**
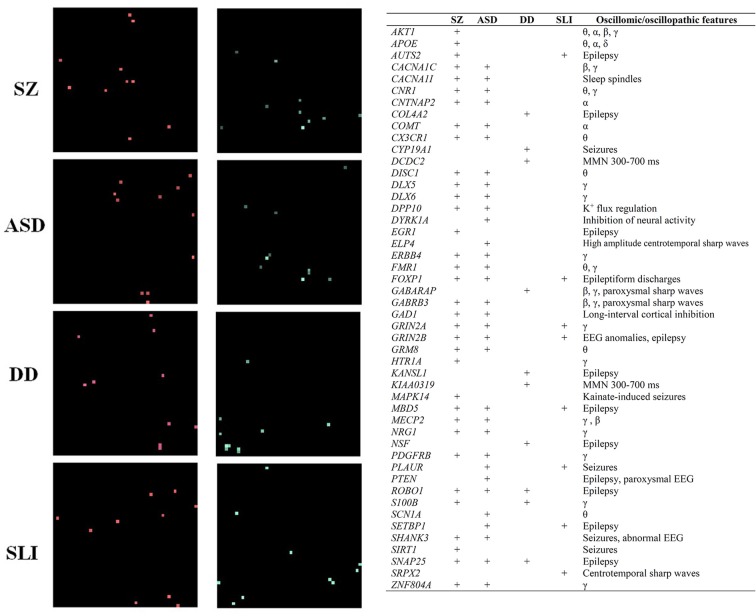
The “Why” Question. Genes involved in brain rhythmicity are expected to exhibit a disorder-specific expression profile in the brain of affected people. The figure shows the expression grids of genes involved in brain rhythmicity that are also candidates for four prevalent conditions involving problems with language: schizophrenia (SZ), autism spectrum disorder (ASD), developmental dyslexia (DD) and specific language impairment (SLI). The grids were generated with Enrichr (amp.pharm.mssm.edu). Each grid square represents a brain region in the Allen Brain Atlas (portal.brain-map.org). Brain regions where genes of interest are upregulated are displayed in red (the brighter the square, the more upregulated the gene is in the selected region). Regions in which genes are downregulated are shown in green (the brighter the color, the more downregulated the gene is). The genes considered in the analysis, which can be regarded as robust candidates for the language oscillogenome, are displayed in the table, with an indication of their role in brain rhythmicity (adapted from Murphy and Benítez-Burraco, 2018a; Figure 2 and Table 1).

Brain oscillations are highly heritable traits (van Beijsterveldt et al., 1996; Linkenkaer-Hansen et al., 2007; Müller et al., 2017), including oscillations related to language (Araki et al., 2016). As Figure 3 proposes, a comprehensive model linking oscillations to neural wiring should be our goal. Murphy and Benítez-Burraco (2018a) show that the basic aspects of the language oscillome (that is, the particular phasal and cross-frequency coupling properties of neural oscillations involved in, and accounting for, language) result from genetic guidance, and a confident list of candidate genes for this guidance can be posited (although see Soloduchin and Shamir, 2018 for an alternative account through which oscillatory activity can emerge *via* an unsupervised learning process of spike timing-dependent plasticity). Moreover, a number of linking hypotheses connecting particular genes and oscillatory behavior implicated in language processing can be posited, suggesting that much of the oscillome is likely genetically-directed; the set of genes implicated here is termed the *oscillogenome* (see also Figure 4 below). Importantly, these candidate genes map on to specific aspects of brain function, particularly on to neurotransmitter function, and through dopaminergic, GABAergic and glutamatergic synapses (see also Koch et al., 2016 for a neuropharmacological review of oscillations).

## Who: Brain Oscillations and Language Disorders

Most cognitive disorders entail problems with language. But there are many differences here depending on who we are focusing our attention on. Whereas each disorder can be said to exhibit a disorder-specific abnormal language profile (with deficits in the domains of phonology, syntax, semantics, or language use), each particular deficit is commonly found in several disorders, to the extent that most of them are shared by different disorders with a different symptomatology and etiology. This accounts for the frequent comorbidity of disorders. Moreover, these deficits are only indirectly related to (broad) cognitive deficits (for instance, certain syntactic deficits involve much broader working memory and attentional problems; Tilot et al., 2015). Finally, although most of these conditions have a genetic basis, the same gene can contribute to more than one cognitive disorder. This circumstance seemingly explains why the divide between the genetics and pathophysiology of prevalent cognitive/language disorders like autism spectrum disorder, schizophrenia or developmental dyslexia remains open. In recent years, a number of promising directions have emerged for investigating the neural and genetic basis of these disorders. Due to an emerging body of work concerning the oscillatory dynamics of language processing, it has become possible to associate certain features of the language deficit profile of autism, schizophrenia and developmental dyslexia with abnormal patterns of brain oscillations. Likewise, contemporary developments have allowed researchers to explore the genetic basis of particular cellular activity giving rise to oscillatory rhythms in distinct brain regions (e.g., Hancock et al., 2017) which appear to differ from neurotypical behavior in certain populations exhibiting language deficits, and which thereby allow us to make inferences about the likely genetic basis of these disorders.

In a series of related articles (Benítez-Burraco et al., 2016; Benítez-Burraco and Murphy, 2016; Murphy and Benítez-Burraco, 2016; Wilkinson and Murphy, 2016; Jiménez-Bravo et al., 2017; Murphy and Benítez-Burraco, 2018a) it has been shown that the distinctive language deficits found in clinical conditions like autism, schizophrenia and developmental dyslexia can be satisfactorily construed in terms of an abnormal, disorder-specific pattern of brain rhythmicity. Moreover, selected candidate genes for these conditions seemingly account for this abnormal rhythmicity and are differentially expressed in selected brain areas (see Figure 4), conferring a degree of specificity to this set of genes compared to other candidates for language dysfunction in these conditions. Ultimately, the genes encompassing the language oscillogenome are expected to exhibit a distinctive, disorder-specific pattern of up- and down-regulation in the brains of patients. In other words, the molecular signature of each disorder from this oscillogenomic perspective is expected to mostly rely not on the set of genes involved, which are thought to be essentially the same, but on their expression patterns in each brain region, which is hypothesized to be different in each condition. This is expected to contribute to the bridging of genes (with their disorder-specific expression profile) and oscillations (with their disorder-specific rhythmic profile) and language (which is also impaired in a disorder-specific way).

As an example of this systems biology approach to language disorders that relies on brain oscillations, consider autism. Both structural and functional aspects of language are impaired in autism. Approximately one-third of children with autism exhibit difficulties with morphosyntax (Tager-Flusberg and Joseph, 2003) and both adults and children with autism typically use a low number of functional words (Tager-Flusberg et al., 1990). This population also integrates and consolidates semantic information differently from neurotypicals when processing sentences (Eigsti et al., 2011). More specific impairments include problems with relative clauses, *wh*-questions, raising and passives (Perovic and Janke, 2013). These difficulties all speak to a more general deficit in procedural memory. Concerning the oscillatory basis of these deficits, increased γ power has been documented for individuals with autism (e.g., Kikuchi et al., 2013), and since this rhythm is involved in the binding of semantic features this finding can likely contribute to a causal-explanatory oscillatory model of language deficits. Kikuchi et al. (2013) additionally found reduced cross-cortical θ, α and β in the brain of individuals with autism, while Bangel et al. (2014) documented lower β power during a number estimation task. Given the role of these slower rhythms in cross-cortical information integration, and the major role β likely plays in syntactic processing (Murphy, 2018), problems with executing complex syntactic operations like passivization and interpreting *wh*-dependencies seems not too surprising. At the same time, many of the differences in cognition and behavior found in autism are seemingly explained by differences in oscillatory activity resulting from pathogenic genetic diversity, mostly in genes indirectly or directly related to GABAergic activity, like *MECP2* (Liao et al., 2012), and genes encoding some of the GABAA-receptor subunits (particularly of β2 and β3; Porjesz et al., 2002; Heistek et al., 2010), or *PDGFRB* (Nguyen et al., 2011; Nakamura et al., 2015).

These oscillatory anomalies found in cognitive disorders in tandem with an increasingly sophisticated oscillatory model of language (see “How: Oscillations and the Linguistic Brain” section above) can yield predictions about the cortical profile of particular individuals exhibiting certain language deficits. Specifically, considering language disorders as “oscillopathic” traits (that is, involving abnormal patterns of brain rhythmicity) is a productive way to generate endophenotypes of the disorders and achieve earlier and more accurate diagnoses.

## Why: Oscillations and Language Evolution

As discussed above, language is a complex system. Accordingly, and addressing the looming question of why our brains alone possess the capacity for language, we should expect that specific evolutionary changes in components of this complex system prompted the transition to language-readiness. At present, we have precise characterizations of the recent evolutionary changes in our brain and in our genetic endowment that seemingly account for our language-readiness (see Boeckx and Benítez-Burraco, 2014; Neubauer et al., 2018; Gunz et al., 2019). Nonetheless, as noted above, brain anatomy and functional maps can only provide indirect and rough accounts of how the brain processes language. Moreover, because the specificity of language can seemingly be hosted at the oscillomic level, and because each species-specific pattern of brain coupling builds on a shared set of basic rhythms, we should expect that the human-specific pattern of coupling accounting for our language-readiness resulted from selected changes in the oscillatory signature of the hominin brain. These modifications can be traced *via* comparative studies, with humans exhibiting a species-specific richness in possible cross-frequency couplings (for references and discussion, see Murphy, 2018). Nevertheless, we should stress that these traces are not thoroughly well-established, and we must rely purely on current understanding.

**Figure 5:**
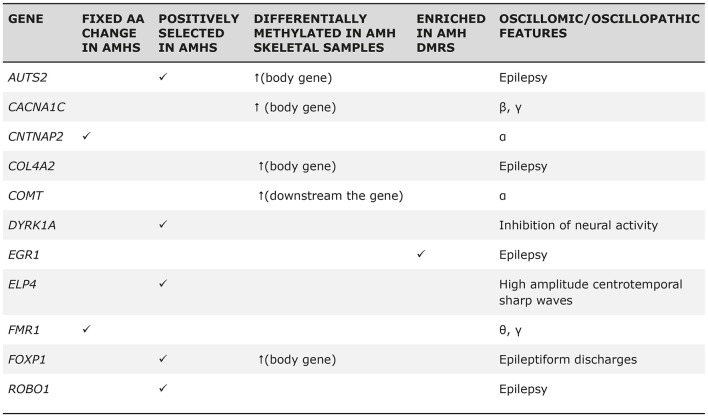
The “Why” Question. Selected genes encompassing the language oscillogenome exhibit fixed derived changes in modern humans compared to extinct Neanderthals, either in their regulatory or coding regions, or in their methylation patterns (suggestive of differences in their expression levels; reproduced from Murphy and Benítez-Burraco, 2018b; Table 1).

Regarding extinct hominins, such as Neanderthals or Denisovans, it is evident that we cannot track the oscillatory activity of their brains. However, it is possible to rely on available (although still scarce) information from genes encompassing the language oscillogenome—as characterized above—to infer the particular changes in phasal and cross-frequency coupling properties of neural oscillations that resulted in the emergence of core features of language. Accordingly, as Figure 5 depicts, several candidates for the language oscillogenome show differences in their methylation patterns (and hence, in their expression levels) between Neanderthals and anatomically-modern humans (we refer the reader to Murphy and Benítez-Burraco, 2018a). Some of these differences can confidently be related to neural function (i.e., directly impacting firing patterns), whereas others have (so far) simply been associated with particular conditions. These differences can be informative of differences in cognitive functions important for language; for instance, we can infer that the working memory capacity of Neanderthals likely differed from that of modern humans due to the differences in θ and γ expression (Murphy and Benítez-Burraco, 2018a). Nevertheless, while they gesture towards a concrete research programme, these suggestions remain highly speculative.

## When: An Eco-Evo-Devo Approach to Language

A growing body of evidence suggests that regions of the human genome showing signals of positive selection in our species are enriched in candidates for cognitive conditions entailing problems with language, like autism (Polimanti and Gelernter, 2017) or schizophrenia (Srinivasan et al., 2016). These findings suggest that these conditions may have mainly developed recently in our evolutionary history. This is seemingly due to the fact that the most recently evolved components of human cognition are more sensitive to the deleterious effect of developmental perturbations resulting from factors either internal to the organism or external to it. This is because of the lack of robust compensatory mechanisms to any damage; these mechanisms are typically found in more ancient biological functions which have been shaped by stronger selective pressures (see Toro et al., 2010 for discussion). In a similar vein, when searching for the basis of genomic trade-offs potentially involved in the evolution of the human brain, Sikela and Searles Quick (2018; p. 2) have concluded that changes in the genome producing beneficial results might persist despite their ability to also produce diseases and that “the same genes that were responsible for the evolution of the human brain are also a significant cause of autism and schizophrenia”. This is in line with current views of complex diseases as the consequence of the uncovering of cryptic variation resulting from the assorted changes (genomic, demographic, behavioral) promoting the transition from an ape-like biology to a human-specific biology.

As noted above, a systems biology approach to language is preferable since it allows us to understand how language emerges from the complex interactions among thousands of biological factors, most notably oscillations. It is now clear that because language evolved mostly as a result of specific changes in the developmental path of the hominin brain in response to changes in the environment in which our ancestors lived (the latter encompassing both physical and cultural factors), we need to consider developmental, evolutionary, and ecological aspects on a par. This can be viewed as an *eco-evo-devo* approach to language, that pays attention to language evolution (*evo*) and human ecology (*eco*) to better understand language development (*devo*; see Benítez-Burraco and Kimura, 2019). This approach should enable us to improve our understanding of how language is implemented in the brain, how it evolved, and how it is disrupted in language disorders. In addition, the evidence presented suggests that this can be ideally achieved by focusing on oscillations, in particular since oscillations can explicitly be linked, in some way, to *all* major topics in the study of the computational nature of language (i.e., online processing; timing of evolution; brain mapping; explanation for language deficits; development). Specifically, oscillations might be a better (or perhaps, the optimum) candidate for properly defining the morphospace or adaptive landscape of language growth in the species, either pathological or neurotypical; that is, defining the limited set of language faculties available during development.

## Conclusions

Overall, the evidence presented in this article suggests that brain oscillations can be a very fruitful approach for understanding how language is implemented in our brain as a result of our evolutionary history. This is not just because oscillations are both domain-general and domain-specific, but because they help explain why and how *processing*, *evolution* and *development* are closely interwoven. Yet, we should stress that while oscillations are improving our understanding of the neural basis of language, they are not (currently) improving our understanding of the language system itself: programmatic and experimental direction from the theoretical linguistics literature will still be required, and care should be taken when attributing certain properties of “language” (however formulated, e.g., computational system, externalization system) to particular oscillatory behavior. Although new avenues for research are rapidly opening up, there remain a large number of unanswered questions: Which sub-domains of linguistics have the potential to make greater contact with the life sciences (e.g., pragmatics)? What are the anatomical similarities and differences regarding human and nonhuman temporal processing networks? How does the notion of a traveling oscillator tie in with existing findings concerning the supposedly fixed, regionalized oscillatory activity found in existing EEG and MEG experiments of language processing? How might one test the hypothesis that nonhuman primates exhibit a distinctly organized array of cortical cross-frequency couplings? Solving these and other complex questions will help refine our oscillatory view of human language.

## Author Contributions

Both authors contributed to all sections.

## Conflict of Interest Statement

The authors declare that the research was conducted in the absence of any commercial or financial relationships that could be construed as a potential conflict of interest.
